# Gold Clusters: From the Dispute on a Gold Chair to the Golden Future of Nanostructures

**DOI:** 10.3390/molecules26165014

**Published:** 2021-08-19

**Authors:** Maria Luisa Ganadu, Francesco Demartin, Angelo Panzanelli, Ennio Zangrando, Massimiliano Peana, Serenella Medici, Maria Antonietta Zoroddu

**Affiliations:** 1 Department of Humanities and Social Sciences, Università degli Studi di Sassari, Via Roma 151, 07100 Sassari, Italy; 2 Department of Chemistry, Università degli Studi di Milano, Via Camillo Golgi, 19, 20133 Milan, Italy; francesco.demartin@unimi.it; 3 Department of Chemistry and Pharmacy, Università degli Studi di Sassari, Via Vienna 2, 07100 Sassari, Italy; panza@uniss.it (A.P.); peana@uniss.it (M.P.); sere@uniss.it (S.M.); 4 Department of Chemical and Pharmaceutical Sciences, Università degli Studi di Trieste, Via L. Giorgieri 1, 34127 Trieste, Italy; ezangrando@units.it

**Keywords:** gold complexes, gold clusters, transition metal complexes, phosphine ligands

## Abstract

The present work opens with an acknowledgement to the research activity performed by Luciana Naldini while affiliated at the Università degli Studi di Sassari (Italy), in particular towards gold complexes and clusters, as a tribute to her outstanding figure in a time and a society where being a woman in science was rather difficult, hoping her achievements could be of inspiration to young female chemists in pursuing their careers against the many hurdles they may encounter. Naldini’s findings will be a key to introduce the most recent results in this field, showing how the chemistry of gold compounds has changed throughout the years, to reach levels of complexity and elegance that were once unimagined. The study of gold complexes and clusters with various phosphine ligands was Naldini’s main field of research because of the potential application of these species in diverse research areas including electronics, catalysis, and medicine. As the conclusion of a vital period of study, here we report Naldini’s last results on a hexanuclear cationic gold cluster, [(PPh_3_)_6_Au_6_(OH)_2_]^2+^, having a chair conformation, and on the assumption, supported by experimental data, that it comprises two hydroxyl groups. This contribution, within the fascinating field of inorganic chemistry, provides the intuition of how a simple electron counting may lead to predictable species of yet unknown molecular architectures and formulation, nowadays suggesting interesting opportunities to tune the electronic structures of similar and higher nuclearity species thanks to new spectroscopic and analytical approaches and software facilities. After several decades since Naldini’s exceptional work, the chemistry of the gold cluster has reached a considerable degree of complexity, dealing with new, single-atom precise, materials possessing interesting physico-chemical properties, such as luminescence, chirality, or paramagnetic behavior. Here we will describe some of the most significant contributions.

## 1. Introduction

**Scheme 1:**
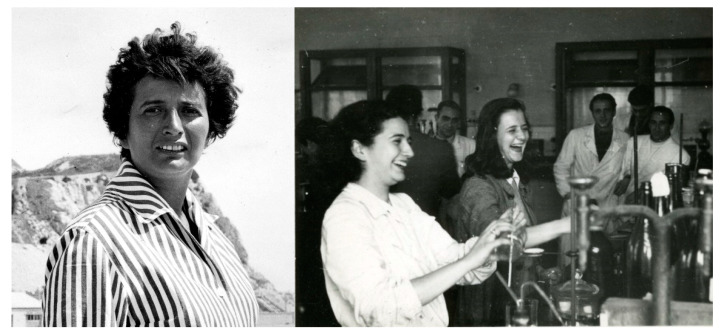
Luciana Naldini (1960) (**left**); Laboratory work in Milan (**right**); Luciana Naldini is in the forefront of the picture.

Luciana Naldini (Scheme 1) was born in 1925 in Pisa, where she graduated with full marks in general chemistry. Immediately after the end of the war, she moved to the University of Milan as an assistant of professor U. Sborgi at the Faculty of General and Inorganic Chemistry, where she remained even when the chair passed to professor L. Malatesta. She taught chemistry for the degree courses of biology, medicine, and geology until 1980, when she was appointed full professor at the University of Sassari, where she also became Dean of the Faculty of Chemistry and Director of the CNR (Consiglio Nazionale delle Ricerche) Institute for the Application of Advanced Chemical Technologies to Agrobiological problems, until 1995, when she passed away following a serious illness. Her seminal work is testified by many scientific papers supported by a sound, critical approach to scientific data. She attended national and international conferences as a speaker and was also a science columnist. She kept reminding that “The Periodic Table belongs to everyone” every time a colleague made her observe that someone imitated her compounds.

This contribution, in addition to being a tribute to Luciana Naldini as a researcher, would also like to underline the difficulty women encountered in reaching leadership positions in all spheres of life, and science is not an exception. This lack of gender equality conditions in a scientific institution, which should be founded on the recognition of one’s merit, competencies, and creativity, regardless of other personal features and orientations, deeply clashes with the traditional view of science. Despite this situation, she was appointed Full Professor and Director of the CNR Institute when she moved to the island of Sardinia, where she was well-liked among colleagues and students and developed her independent field of work.

During the early years of research in Milan, Luciana Naldini synthesized a series of gold clusters [1,2] comprising six to eleven gold atoms that could not be explained by the standard rules of valence and only a few of these have been structurally characterized. Among them, the unique Au_6_ compound exhibits a number of intriguing features with respect to relativistic effects, aurophilicity, and Au^…^Au contacts [3,4,5,6]. These aspects of gold clusters were relatively new, since the first reports of the relativistic effects studied through Dirac scattered-wave calculations dated back to the second half of the 1980s [7,8,9], when also the relationship between aurophilicity and relativistic effects started to emerge in studies carried out on (Ph_3_PAu)_6_ clusters [10,11] similar to those prepared by Naldini. Such clusters were first reported in a thesis work dating back to 1994 [12], of which she was supervisor, and gave additional light in this field, but they also concluded a period of lively research activity, carried out in collaboration with her esteemed coworkers, starting from L. Malatesta, recognized as the father of gold clusters chemistry [13], together with F. Cariati, M. Manassero, G. Simonetta, and many others that were involved in this exciting exploration of the chemistry of gold. Finally, within this fascinating area, we report a view on recent studies on electron count and aurophilic interactions of which Naldini and coworkers laid the first groundwork.

## 2. Results

In her career, Luciana Naldini was particularly interested in the synthesis and structural studies of transition metal complexes, both in solid state and in solution. The metal complexes have been characterized by means of several spectroscopic techniques (NMR, IR, UV-Vis, EPR), XPS, single-crystal X-ray diffraction, as well as potentiometric and electrochemical analyses.

**Figure 1:**
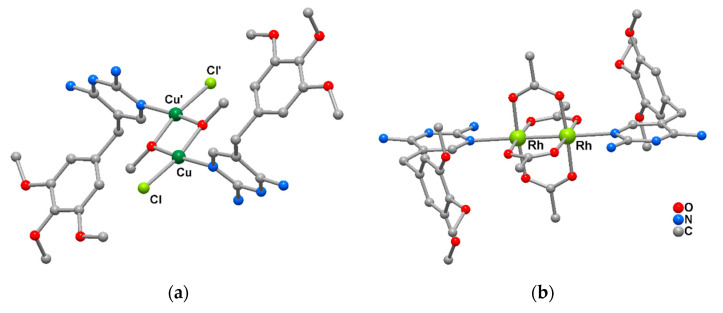
Molecular structure of Cu(II) and Rh(II) complexes with antibiotic trimethoprim molecule: (**a**) from [15]; (**b**) from [16]. (H atoms omitted for clarity).

The research was mainly addressed towards complexes with transition metal ions such as Cu(II), Co(II), Rh(II), and Au(0/I) and ligands of biological interest and therapeutic activity. In this field, dimeric Cu(II) [14,15] and Rh(II) complexes [16] have been synthesized with antibiotic trimethoprim or pyrimethamine neutral molecules that behave as monodentate ligands through a pyrimidinic nitrogen atom toward the metal center. In particular, a species containing a squared Cu_2_(OMe)_2_ core in which the metals are held together by two methoxo bridges (Figure 1a), and a dinuclear rhodium lantern-type complex with acetate bridges (Figure 1b) have been obtained.

In addition, monomeric complexes of Co(II) with trimethoprim have also been synthesized [17], and interesting crystal structures have been determined with aminobenzoate derivative ligands, [Cu(bipy)(p-NH_2_-benzoato)] [18], with *o*- and *p*-nitrobenzoate, [Cu(PPh_3_)_2_(NO_2_-benzoate)] [19], as well as with nitrophenols [14].

**Figure 2:**
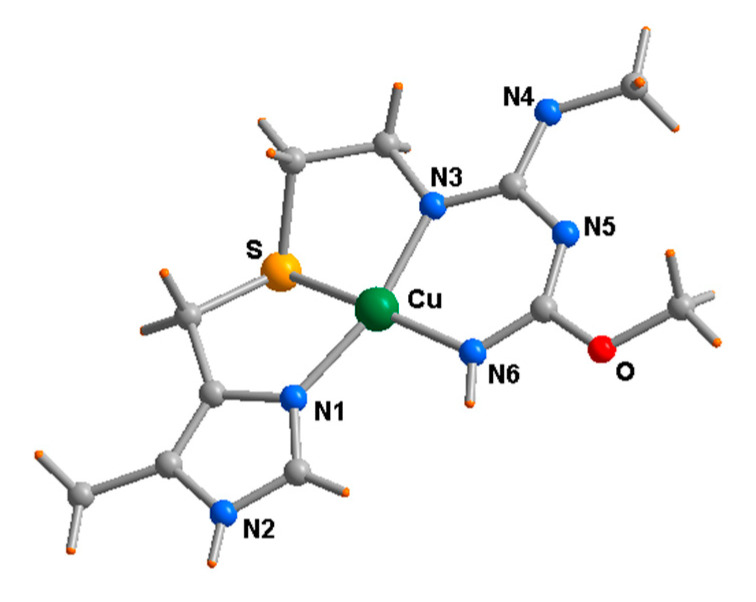
Molecular structure of the square planar cationic copper(II) complex with 5-methylcimetidine modified by methanolic solvolysis of the nitrilic function [21]. (Perchlorate anion not shown for clarity).

These studies were extended to the interaction of histamine (H2)-receptor antagonists, namely the anti-ulcerative drugs cimetidine, famotidine, and ranitidine, with platinum and palladium [20], studied by ^1^H-NMR and potentiometric titration, but also with copper [21], of which the structural study indicates the tetradentate ligand wrapped around the almost square planar metal center (Figure 2).

**Figure 3:**
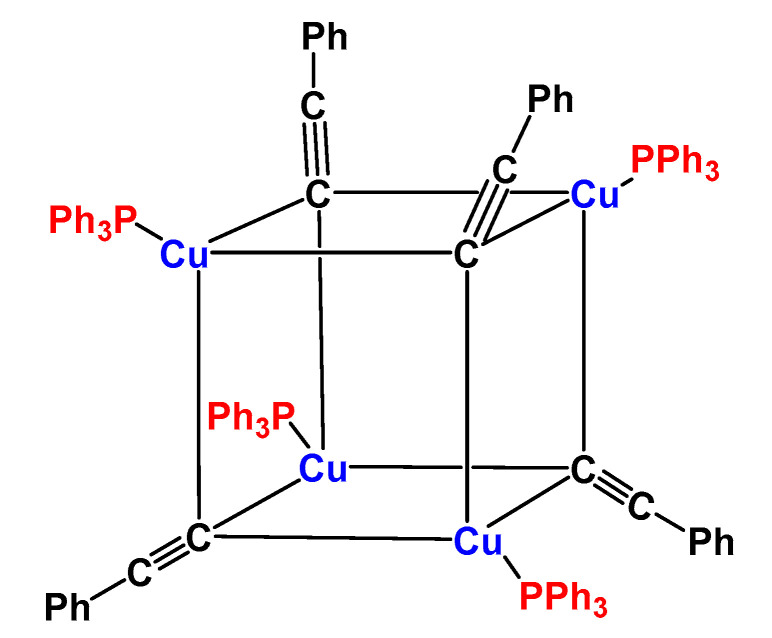
Scheme of the [Cu(PPh_3_)(C≡CPh)]_4_ tetramer complex [22].

It is interesting to note that the use of phosphine ligands has been often a distinctive feature of her studies. The cubane-like [Cu(PPh_3_)(C≡CPh)]_4_ tetramer was obtained by treatment of [Cu(PPh_3_)_2_BH_4_] with phenylacetylene and KOH (molar ratio 1/1/1) in benzene/benzyl alcohol medium. It consists of a tetrahedral skeleton of metal atoms bonded to four terminal phosphine molecules and to four μ-3-bridging phenylacetylide ligands which behave essentially as two-electron donors (Figure 3) [22].

However, the study of gold clusters represents a significant part of the research work of Naldini and coworkers, which started at the University of Milan where gold-phosphine clusters were first reported in 1965 by reacting PPh_3_AuCl with sodium borohydride [23]. It is worth noting that in previous experiments with copper and silver complexes, the [(PPh_3_)_2_M(BH_4_)] (M = Cu, Ag) species were formed, while gold complexes manifested a different behavior, leading to a red crystalline precipitate. At the time, the Au_5_(PPh_3_)_4_Cl^.^5H_2_O formulation was believed for this red compound [24]. It was not apparent until the partial crystal structure of [Au_11_(PPh_3_)_7_(SCN)_3_] was reported in 1969 [25] that the structures of the species formed were more complex than formerly believed and comprising gold(0)-gold(I) bonds. Following these results, the research in this field was expanded by using the diphenyl ethyl phosphine to obtain [(PPh_2_Et)_2_Au_3_Cl^.^H_2_O]_4_ and [(PPh_2_Et)_4_Au_6_Cl]Y (Y = ClO_4_, PF_6_, BPh_4_), [26] thus leading to novel cluster structures.

Since then, many gold cluster compounds have been prepared using diphosphines, for example reduction of [(AuCl)_2_(dppe)] by NaBH_4_ in ethanol gave the [Au_6_(dppe)_2_Cl_2_] cluster [26], and reduction of [Au(L)(NO_3_)] featured [Au_9_L_8_]^3+^, where L = PPh_3_ or P(p-tolyl)_3_ [27,28].

**Figure 4:**
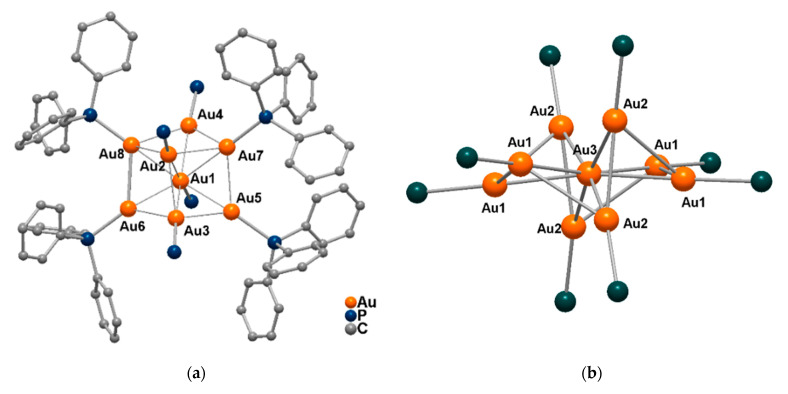
(**a**) Molecular structure of the [Au_8_(PPh_3_)_8_]^2+^ core [30] (only PPh_3_ phosphorous atom are shown in the central part, dializarinsulphonate counterions not shown). (**b**) Molecular structure of the cationic [Au_9_(PPh_3_)_8_]^3+^ core [31] (for sake of clarity phenyl rings of PPh_3_ not shown).

The accurate structure of the [Au_9_(PPh_3_)_8_]^3+^ cluster exhibits a crystallographic *D*_2_ and approximate molecular *D*_2*h*_ skeletal symmetry derived from an icosahedron (Figure 4). The compound has been extensively studied, as the reasons for research being carried out on this specific cluster are the availability and low cost of PPh_3_ along with the ease of cluster synthesis. In fact, this species, deposited and activated on TiO_2_ and SiO_2_, has been explored in catalytic reactions [29] and can undergo transformations to form additional clusters.

Indeed, a few years later, [Au_9_(PPh_3_)_8_]^3+^ was treated with an excess of phosphine in methanol and precipitated as [Au_8_(PPh_3_)_8_](aliz)_2_, (aliz = dializarinsulphonate), by addition of diethyl ether, thus giving rise to a new class of gold cluster compounds [30]. The molecular structure of the cluster shows a centered gold atom connected to seven Au(PPh_3_) moieties [31].

**Figure 5:**
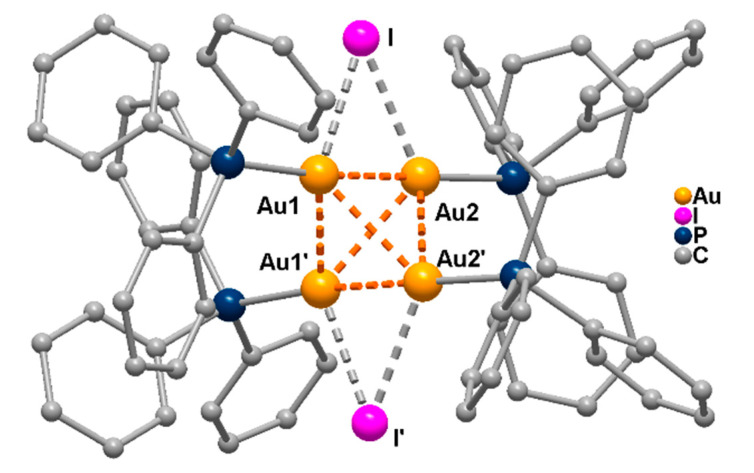
Molecular structure of [Au_4_(PPh_3_)_4_(µ-I)_2_], (from [32]; H atoms omitted for clarity).

[Au_9_(PPh_3_)_8_]^3+^ was also reacted with KI in acetone to give [Au_4_(PPh_3_)_4_(µ-I)_2_], the first tetranuclear gold structure to be reported. The molecular structure shown in Figure 5 presents a crystallographic C_2_ symmetry with two edge bridging iodine ligands at opposite edges of the metal tetrahedron [32].

**Figure 6:**
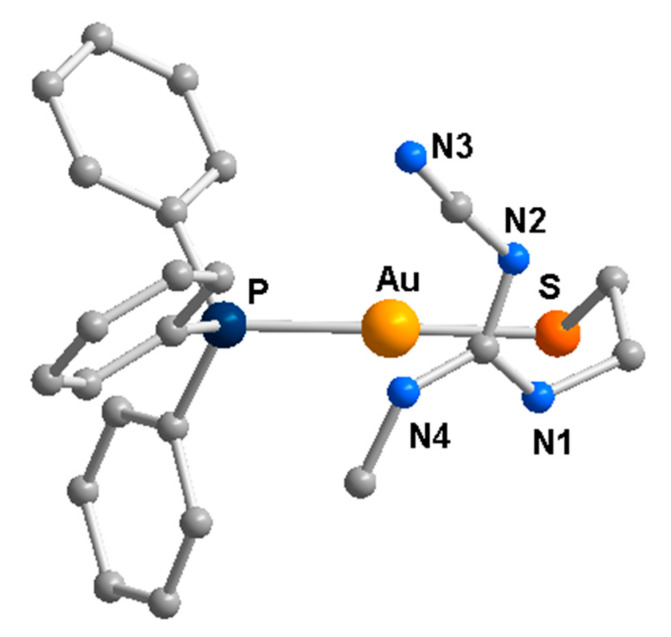
The PPh_3_ gold complex obtained with the modified cimetidine ligand [35].

A study in solution was also conducted on (PPh_3_)Au(C≡CPh) complexes by NMR, indicating that contrary to the solid state findings [33], the existence of Au^…^Au contacts in solution can be excluded, being solvation effects likely stronger than intermolecular gold contacts [34]. In another experiment, the reaction of (PPh_3_)AuCl with cimetidine in an alkaline medium under mild conditions led to a complex (Figure 6), comprising a thiolato ligand resulting from cleavage of one of the thioether bonds of cimetidine [35], thus demonstrating a different behavior by the heavier gold atom with respect to copper (see above).

In order to elucidate the mechanism of the observed C-S bond cleavage, Naldini and coworkers reacted (PPh_3_)Au(NO_3_) with benzyl phenyl sulfide (PhCH_2_-S-Ph) in KOH at ambient temperature. The X-ray diffraction study of the obtained product, as a BF_4_^−^ salt, revealed the formation of the cluster [(PPh_3_)_6_Au_6_O_6_]^2+^ as having a chair configuration and crystallizing in a unit cell of comparable parameters as those reported [36]. Complexes with the hexanuclear Au_6_L_6_ core, where gold atoms adopt the edge-shared bi-tetrahedral structure and in an average oxidation state between 0 and 1, have been widely studied, as for example [Au_6_(PPh_3_)_6_](NO_3_)_2_ [37], [(AuPPh_3_)_6_(NNR_2_)_2_] with R = Me, or Ph [38], and [Au_6_(dppp)_4_](NO_3_)_2_ [39].

**Figure 7:**
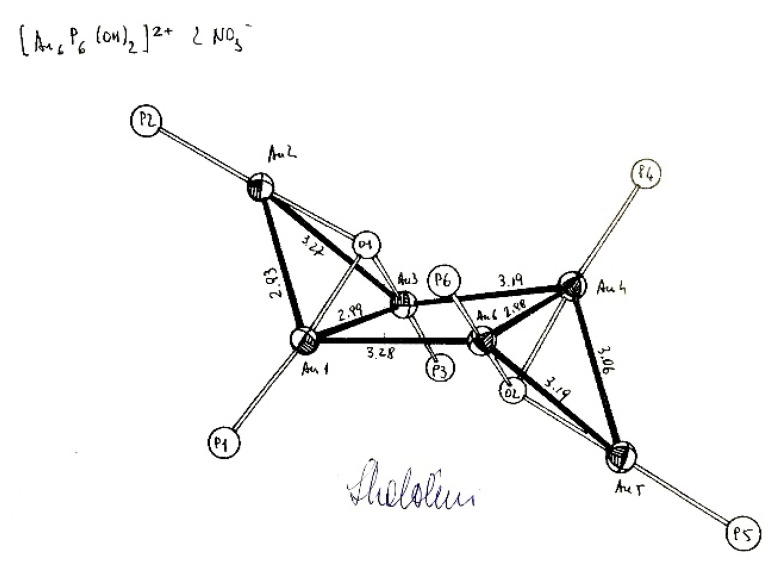
Naldini’s notes on the [(PPh_3_)_6_Au_6_(OH)_2_]^2+^ cluster with her signature (from ref [12]).

The [(PPh_3_)_6_Au_6_(OH)_2_]^2+^ cluster, (Figure 7), synthesized by Naldini was reported in her student’s thesis [12], but single crystals were of poor quality for suitable X-ray structural analysis. However, it was observed that: (i) all the Au^…^Au distances were within the same order of magnitude; (ii) a prolonged reaction at higher temperature (>50 °C) led to a dark orange-red solution, characteristic of polynuclear gold clusters, indicating the possible partial reduction of gold to Au(0), the number of coupling being determined by kinetics factors.

**Scheme 2:**
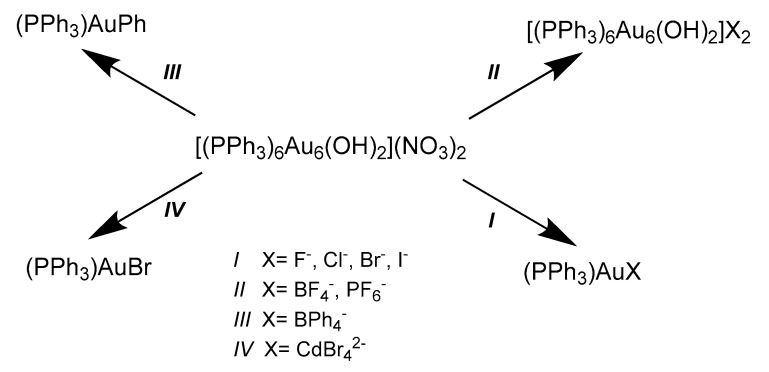
Reaction scheme of the complex [(PPh_3_)_6_Au_6_(OH)_2_](NO_3_)_2_ with different anions.

With this in mind, the presence of two Au(0) atoms out of six was postulated, with the presence of two OH^-^ anions replacing the oxo groups indicated by other research groups [36,40], since the energy gain due to the two s^1^ electrons should compensate for the repulsion of ions of the same charge. Numerous exchange reactions were performed in order to obtain crystals of good quality, and the derivatives with NO_3_^-^ and BF_4_^-^ counterions were structurally characterized by single crystal X-ray diffraction analysis. However, in some cases, the nucleophilic anions led to the correspondent mononuclear (PPh_3_)AuX complex (Scheme 2). The organometallic complex (PPh_3_)AuPh, obtained by extraction of phenyl group from the B(Ph)_4_^-^ anion, was structurally characterized by X-ray diffraction by Hong et al. [41].

The presence of hydroxyl groups in [(PPh_3_)_6_Au_6_(OH)_2_](NO_3_)_2_ cluster was supported by a series of detailed experiments [12]. The ^1^H NMR spectrum in CD_2_Cl_2_ showed a complex pattern centered at 7.45 ppm for the phosphine protons and a singlet at ca. 2 ppm, with a 90:2 intensity ratio. Due to possible errors in the OH assignment for the great disproportion of the number of protons, the test was comforted by the subsequent addition of D_2_O that led to the disappearance of the peak at 2 ppm. The ^17^O NMR spectrum showed three singlets at −12.312, 6.048 and 28.080 ppm, which can be assigned to water, ethanol (as the internal standard), and OH^-^ groups, respectively. A coulombometric reduction of a solution of [(PPh_3_)_6_Au_6_(OH)_2_](NO_3_)_2_, performed at −1.50 Volt vs. SCE, involved the consumption of 4.05 electrons/mol; a result likely confirming the presence of four Au(I) atoms out of six. Mass spectrometry revealed peaks at 2789 (calcd 2789.6) and 1394 (calcd 1394.7) for the cations [(PPh_3_)_6_Au_6_(OH)_2_]^+^ and [(PPh_3_)_3_Au_3_(OH)]^+^, respectively, and no peak at 1393 correspondent to [(PPh_3_)_3_Au_3_O]^+^ [12].

In addition, the XPS characterization [42] of the electronic structure of the cluster under study showed two peaks in the region of the 4f gold electrons, at 85.8 (for Au(I)) and 83.9 eV (for Au(0)) associated to a peak area of 2:1 (thus to a 4:2 ratio). Three peaks, at 531.5, 532.7, and 530.2 eV, were found in the 1s oxygen electron region, assigned to the OH, nitrate, and diphenyl ether groups.

The analogous complex having SH groups replacing the hydroxyl ones was also prepared by Naldini, [(PPh_3_)_6_Au_6_(SH)_2_](NO_3_)_2_ [12]. It is worth noting that Naldini and coworkers prepared plenty of similar gold clusters, also with mixed-metals, for which no structural information is available. At that time, the lack of an easy X-ray facility or the difficultly to obtain suitable crystals for diffraction analysis delayed the development of the study, and the structure, as well as the formulation of these complexes, could be only hypothesized. In support of these hypotheses, the presence of µ^3^-OH species in gold cluster cations and their equilibria in wet solution have been reported a few years ago [43].

**Figure 8:**
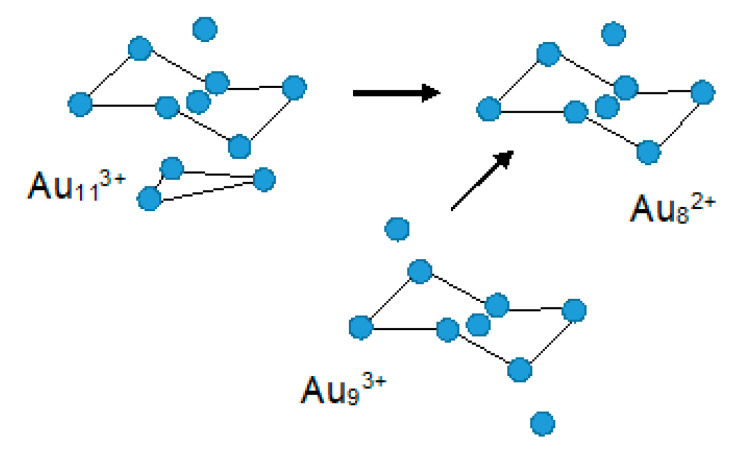
The Au_8_, Au_9_, and Au_11_ clusters comprising a central chair-like hexagon [30].

All the Au_8_, Au_9_, and Au_11_ clusters show a central chair-like hexagon, thus the Au_6_L_6_ system can be considered as a starting building block for the construction of clusters of higher nuclearity [30] (Figure 8).

## 3. Recent Advances

In the last years, after several decades have passed since the outstanding work from Naldini and coworkers, the chemistry of gold clusters has reached a notable degree of complexity, dealing with new materials possessing interesting physico-chemical properties, such as luminescence [44,45,46,47], chirality [48], or paramagnetic behavior [49]. In the mare magnum of gold complexes, clusters, nanoclusters, and nanoparticles so numerously reported in the literature, which have already been reviewed [50,51,52,53], we decided to focus the second part of this paper on some recent compounds with remarkable features, to briefly highlight the progress made in this field, the latest achievements, and above all the current trends in research.

### 3.1. Superatom Gold Cluster Complexes

The most recent studies range from small molecular-like species to larger nanoparticle-like clusters with hundreds of metal atoms. Contrary to classical nanoparticles, such nanoclusters, with a core size smaller than 2 nm, have a definite molecular structure and thus a precise molecular formula, of the [Au_n_L_m_]^z^ kind (where L is the protecting ligand, and z is the net charge of the metal) [54], conferring them molecule-like physical and chemical properties. Meanwhile, the electronic shell closing, geometry, and the staple motif can be tuned with single-atom precision.

Special attention has been posed to the stabilization and protection of Au clusters, as the outermost layer of these structures interacts with the external environment, determining their characteristics (e.g., chirality, optical absorption, photoluminescence, hydrodynamic size, etc.) and the possibility of application in different research areas, for example, electronics [55,56], catalysis [57,58,59], and the biomedical field (drug and gene delivery, biosensors, photothermal and photodynamic therapy, imaging, cancer treatment, etc.) [60,61,62,63,64].

Among the commonly employed ligands for monolayer-protected gold nanoclusters, phosphines were historically the most frequently used, leading to Au clusters of various sizes and structures. More recently (especially in the past two decades), thiolates (SR) have emerged as precious ligands in the preparation of Au_n_(SR)_m_ clusters, because the strong sulfur-Au bonds and the aurophilic interactions among gold atoms determine their high stability [50,65,66] in solution and subsequently their possible applications. Other organic ligands that have been used to produce atomically precise Au clusters are selenolates, tellurolates, N-heterocyclic carbenes (NHCs), and alkynes [54].

Gold is a suitable metal to build stable nanostructures, although small clusters tend to be more reactive. Their shape can vary greatly, from the flat flakes of clusters with less than 12 atoms to spherical or spheroidal structures when the overall number of valence electrons is equal or close to a “magic” value, given by the n* = 2(L + 1)^2^ rule, where L is an integer. The relative values (2, 8, 18, 32, 50, 72, etc.) coincide with the number of electrons necessary to fill the orbitals in the electron shells of an atom, and in fact, a spherical gold cluster can be considered as a noble-gas superatom [67], leading to closed-shell configurations: [S^σ^]^2^; [S^σ^]^2^[P^σ^]^6^; [S^σ^]^2^[P^σ^]^6^[D^σ^]^10^; etc. As evidence, clusters with 18 electrons (L = 2) with filled S, P, and D molecular orbitals are remarkably robust [68]. When Au clusters adopt non-spherical geometries the degeneracy of the P^σ^ shell is removed, leading to [S^σ^]^2^[P^σ^]^2^ and [S^σ^]^2^[P^σ^]^4^ configurations for prolate and oblate distortions, respectively, due to the high energy gap among the P molecular orbitals which favors the stabilization of closed sub-shell electronic structures. Examples of this are the gold clusters with phosphines as the ligands: [Au_4_(P^t^Bu_3_)_4_]^2+^ of spherical shape ([S^σ^]^2^), [Au_6_(PPh_3_)_6_]^2+^ and [Au_6_(dppp)_4_]^2+^ prolate ([S^σ^]^2^[P^σ^]^2^), [Au_7_(PPh_3_)_7_]^+^ oblate ([S^σ^]^2^[P^σ^]^4^) [13] are representative examples of gold clusters with phosphines ligands that verify this simple analysis.

Most of the reported superatom clusters are heterometallic gold structures “doped” with other noble metals (Ag, Cu, Pt, Pd, Ir, etc.) (see for instance [69]) or intermetallic species; nevertheless, several homometallic gold clusters stabilized by different ligands have been published. In this case, the “magic number” may vary according to the electron-withdrawing ability of the ligands, and is better described by the formula n* = Nv_X_ − M − z, where N is the number of core metal atoms (X), v_X_ the atomic valence, M the number of electron-localizing (or electron-withdrawing) ligands, and z the overall charge on the complex. Thus, extraordinary stability is associated with a total electron count of n* = 2, 8, 18, 34, 58, 92, 138… [67]. Occasionally, 20 and 40 electrons can also lead to stable clusters [70].

Relatively to low n*, Au_13_ superatom complexes protected by N-heterocyclic carbene ligands were studied, showing that their structures are formed by an icosahedral Au_13_ core with nine NHC ligands and three chlorides symmetrically arranged around [71]. This arrangement favors the formation of multiple CH-π and π-π interactions, as confirmed by crystallographic analyses, which stiffen the ligand and probably promote the extraordinarily high photoluminescent quantum yields (vide infra) of these complexes (up to 16.0%, a value which is remarkably higher than that of the most luminescent ligand-protected Au_13_ clusters). DFT calculations suggest an 8-electron superatom configuration for these kinds of complexes.

A slightly different structure with a closed electronic shell (again 8 electrons) is represented by the hydride-doped gold superatom (Au_9_H)^2+^, a nearly spherical cluster, as revealed by DFT calculations, obtained by inserting a hydride anion into [Au_9_(PPh_3_)_8_]^3+^, which is a 6-electron oblate gold cluster with a coordinatively unsaturated site, to give the 8-electron [Au_9_H(PPh_3_)_8_]^2+^ species [72]. The H^−^ anion with its two 1s electrons contributes to the superatomic electron count, giving a [S]^2^[P]^6^ configuration. The hydride can be displaced by an Au-Cl fragment, and the subsequent addition of a second Au-Cl group leads to the formation of the Au_11_^3+^ cluster, with the same number of valence electrons.

Transformation of a non-superatom into a superatom nanocluster was obtained also in the case of the thiolate-protected cluster [Au_23_(SC_6_H_11_)_16_]^−^ which was converted into Au_36_(TBBT)_24_ (TBBT = 4-*tert*butylbenzenethiolate) via Au_28_(TBBT)_20_ formation, by using the ligand exchange-induced size/structural transformation (LEIST) approach [73].

An analogous nanocluster, Au_44_(2,4-DMBT)_26_ (2,4-DMBT = 2,4-dimethylbenzenethiolate), is an 18 electron superatom possessing chiral properties, conferred by the peculiar arrangement of staples (bridging thiolates) and part of the Au_29_ kernel atom [74].

### 3.2. Chiral Gold Cluster Complexes

The presence of a chiral element on a gold cluster can determine its potential applications in a number of fields, for instance, asymmetric catalysis, chiral recognition, or in chiroptical materials [75,76]. Such an element of chirality can be found either in the gold core or in the capping ligands, or both [77].

In the first case, (i) the structure of the metal skeleton is intrinsically chiral, or (ii) an inherently achiral core with a chiral shell is created by the symmetric disposition of surface-protecting units. Instead, in the latter case, chiral ligands confer chiroptical properties to the Au nanoclusters via vicinal effects or a chiral electrostatic field.

**Figure 9:**
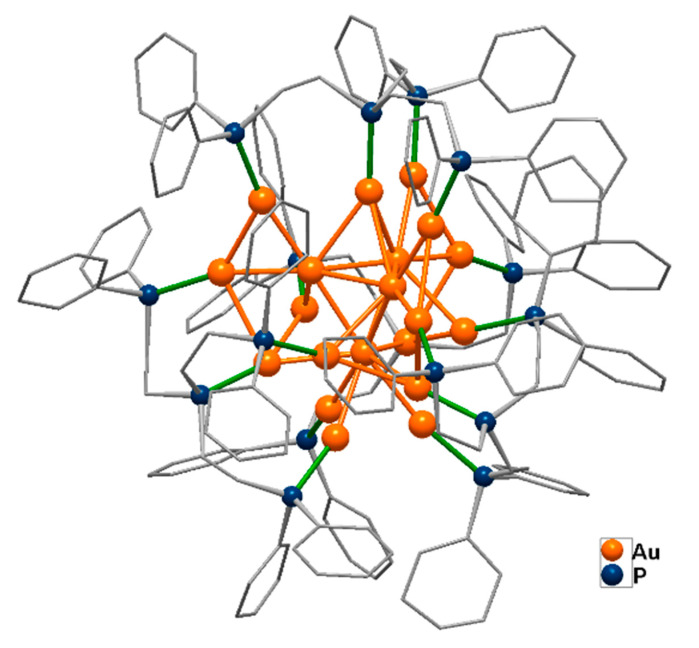
The chiral cation [Au_20_(PP_3_)_4_]^4+^ (PP_3_ = tris(2-(diphenylphosphino)ethyl)phosphine) [78].

When the chiral element is located inside the gold core itself, its inherent or intrinsic chirality is especially enthralling being different in nature with respect to that of conventional chiral molecules. The comprehension and application of optical properties of chiral gold clusters is still a major challenge, due to scarce structural evidence of enantiomerically pure Au nanostructures, with just a very few cases described in the literature. The first reported species of this kind, [Au_20_(PP_3_)_4_]Cl_4_, (PP_3_ = tris(2-(diphenylphosphino)ethyl)phosphine), possesses a C_3_ symmetric molecular structure consisting of a combination of an achiral icosahedral Au_13_ unit and a helical Y-shaped Au_7_ motif, which confers chirality to the whole structure (Figure 9) [78].

More recently, an inherently chiral Au_24_ framework with a nearly spherical shape and high stability due to its 18-electron system was prepared in both its enantiomeric forms by using Chiraphos [2,3-bis(diphenylphosphino)butane] as the chiral auxiliary. It contains a square antiprism-like octagold (essentially achiral), twinned by two helicene-like hexagold motifs [79]. The inherent chirality of the superatom cluster resided in the double-helical hexagold strands induced by the (R,R)- or (S,S)-diphosphines.

Other examples of intrinsically chiral gold nanostructure may be found in the small enantiomeric clusters with formulas [Au_9_(R-/S-BINAP)_4_](CF_3_COO)_3_ and [Au_10_(R-/S-BINAP)_4_(p-CF_3_C_6_H_4_C≡C)](CF_3_COO)_3_ [80]. The Au_9_ core has a C_2_ symmetry and is composed of two Au_4_ tetrahedra sharing an Au atom, and two additional Au atoms attached on the opposite side, which can be considered as a distorted crown structure. Instead, the Au_10_ species has a very low symmetry (C_1_) due to an exterior Au atom attached to one of the corners of a tetrahedron. Both the couples of enantiomers show good chiroptical properties.

### 3.3. Luminescent Gold Cluster Complexes

Luminescence is another characteristic of many gold cluster complexes, which could also be exploited to prepare OLEDs (organic electroluminescent devices) [81]. A typical behavior may be seen in the switch of color caused by the change in the solvent used to prepare a series decanuclear gold(I) μ^3^-sulfido complexes with the generic formula [Au_10_XPh_2_PN(C_n_H_2n+1_)PPh_2_}_4_(μ^3^-S)_4_](ClO_4_)_2_ bearing alkyl chains of various lengths on the aminodiphosphine ligand [82]. These complexes tend to form supramolecular nanoaggregate assemblies upon solvent modulation, whose photoluminescence colors can be switched from green to yellow and to red by using different solvent systems. This, in turn, causes substantial changes in the nanostructured morphology of such clusters, from spherical to cubic shape.

In the past couple of years or so, some luminescent new Au clusters have been prepared, the most interesting being a series of small alkyl thiolato-protected Au complexes with general formula Au_2_(SR)_2_ and Au_3_(SR)_3_ based on the C_4_H_9_SAu (1-butanethiol), C_6_H_13_OSAu (6-mercapto-1-hexanol), C_12_H_25_SAu (1-dodecanethiol) and C_6_H_5_SAu (thiophenol) units [83]. They were formed in a one-pot reduction of tetrachloroaurate in the presence of a threefold excess of the thiolate ligands, and they all show high luminescence under UV light, which is limited to the range 550–700 nm in solution, although a full emission in the range 550-850 nm was recorded for the solids using different techniques.

**Figure 10:**
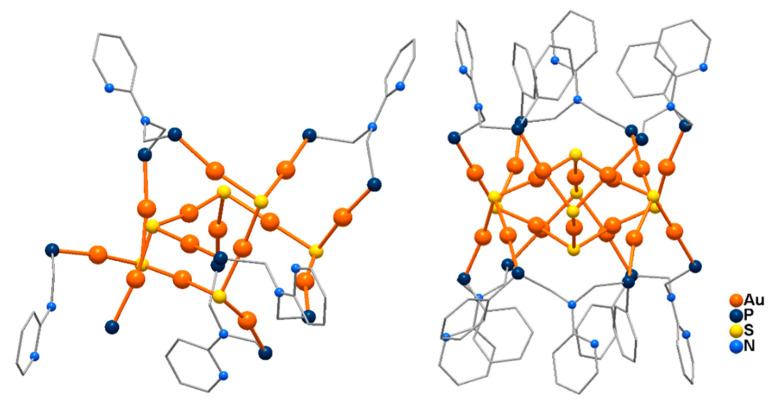
[Au_18_S_8_(bdppmapy)_6_]^2+^ (**left**) and [Au_14_S_6_(bdppmapy)_5_]^2+^ (**right**) cations (bdppmapy = N,N-bis-(diphenylphosphanylmethyl)-2-aminopyridine, phenyl rings bound to P atoms not shown for clarity) [84].

Relatively small polinuclear sulfido Au(I) clusters, with formulas [Au_14_S_6_(bdppmapy)_5_]Cl_2_ and [Au_18_S_8_(bdppmapy)_6_]Cl_2_, where bdppmapy = N,N-bis-(diphenylphosphanylmethyl)-2-aminopyridine (Figure 10), are very stable, strongly luminescent with a high quantum yield, good solubility, low cytotoxicity, and have been exploited as lysosome trackers for live cell imaging [84]. [Au_14_S_6_(bdppmapy)_5_]^2+^ represents a new cluster skeleton-type formed by an Au_6_S_2_(bdppmapy) unit and an Au_8_S_4_(bdppmapy)_4_ crown connected to each other through Au−S and aurophilic interactions. The Au_6_S_2_ unit adopts a cubane structure where the S atoms are located across the body diagonal of the solid, and the Au atoms are bonded to a P atom from the bottom bdppmapy, or capped with an Au_2_S triangle. Each S atom of the cluster adopts a triply bridging coordination mode to bind three Au atoms from the Au_6_S_2_ cubane. The 18 gold atoms in [Au_18_S_8_(bdppmapy)_6_]^2+^ are connected by eight triply bridging S atoms and surrounded by six bdppmapy ligands. Both clusters are air-stable and emit bright yellow-green light (λ_em_ 540 nm and 518 nm, respectively) in the solid state, with corresponding quantum yields of 20.7% and 26.6% at room temperature. Their luminescence greatly enhanced cellular imaging of lysosomes in living cells, with long-term tracking of lysosomes up to 36 h without any evident loss of luminescence intensity.

## 4. Conclusions

Nowadays plenty of papers, which addressed the relationships between the structures of clusters and their skeletal electron counts, have been reported, often citing Naldini’s gold structures. For example, we would like to mention a unified model developed to achieve a fundamental understanding of the stability of ligand-protected gold clusters [85], a theoretical study of the electronic and optical properties of [Au_8_L_8_]^2+^ species (where L = PH_3_, PPh_3_) using a density functional (DFT) approach and the time-dependent density functional theory (TDDFT) [86], the description of triphenylphosphine-stabilized undecagold species [87], as well as a description of geometrical structures and unique optical properties of phosphine-coordinated cold clusters [88], to cite but a few. Recently, it was reported that gold phosphine complexes show anticancer activity towards a panel of different tumor cell lines [89]. In conclusion it is interesting to cite a theoretical approach of bonding patterns in gold clusters [13] where Mingos states that “*The detailed electronic structures of metal clusters will eventually be tackled using increasingly accurate DFT MO calculations, but the presence of conceptual models… will remain valuable for new generations of chemists, providing an important pedagogical introduction to inorganic structural chemistry*”.

## Abbreviations

aliz	dializarinsulphonate
bdppmapy	N,N-bis-(diphenylphosphanylmethyl)-2-aminopyridine
BINAP	2,2′-bis(diphenylphosphino)-1,1′-binaphthyl
Chiraphos	2,3-bis(diphenylphosphino)butane
DFT	density-functional theory
DMBT	dimethylbenzenethiolate
dppe	diphenylphosphinoethane
dppp	diphenylphosphinopropane
EPR	electron paramagnetic resonance
IR	Infra-red spectroscopy
LEIST	ligand exchange-induced size/structural transformation
NHC	N-heterocyclic carbene
NMR	nuclear magnetic resonance
PPh_3_	triphenylphosphine
SCE	saturated calomel electrode
TBBT	4-tertbutylbenzenethiolate
TDDFT	time dependent density functional theory
XPS	X-ray photoelectron spectroscopy

## References

[1] Malatesta L. (1975). Cluster compounds of gold. Gold Bull..

[2] Naldini L., Cariati F., Simonetta G., Malatesta L. (1966). Gold-tertiary phosphine derivatives with intermetallic bonds. Chem. Commun..

[3] Arratia-Pérez R., Hernández-Acevedo L. (1993). Spin-orbit effects on heavy metal octahedral clusters. J. Mol. Str. THEOCHEM.

[4] Häberlen O.D., Chung S.-C., Stener M., Rösch N. (1997). From clusters to bulk: A relativistic density functional investigation on a series of gold clusters Au_n_, *n* = 6,…,147. J. Chem. Phys..

[5] Deka A., Deka R.C. (2008). Structural and electronic properties of stable Au_n_ (*n* = 2–13) clusters: A density functional study. J. Mol. Str. THEOCHEM.

[6] Schmidbaur H., Schier A. (2008). A briefing on aurophilicity. Chem. Soc. Rev..

[7] Arratia-Perez R., Malli G.L. (1986). Bonding in the octahedral Au_6_^2+^ cluster. Chem. Phys. Lett..

[8] Arratia-Pérez R., Malli G. (1986). Dirac scattered-wave calculations for Ag^2+^_3_, Au^q+^_3_, and Au^q+^_4_ (q = 1, 2) clusters. J. Chem. Phys..

[9] Ramos A.F., Arratia-Perez R., Malli G.L. (1987). Dirac scattered-wave calculations on an icosahedral Au_13_ cluster. Phys. Rev. B.

[10] Scherbaum F., Grohmann A., Huber B., Krüger C., Schmidbaur H. (1988). “Aurophilicity” as a Consequence of Relativistic Effects: The Hexakis(triphenylphosphaneaurio)methane Dication [(Ph_3_PAu)_6_C]^2^^⊕^. Angew Chem. Int. Ed. Engl..

[11] Rösch N., Görling A., Ellis D.E., Schmidbaur H. (1989). Aurophilicity as concerted effect: Relativistic MO calculations on carbon-centered gold clusters. Angew Chem. Int. Ed. Engl..

[12] Morelli F. (1994). Una Sedia d’Oro Tenuta Insieme da Due Chiodi? Identificazione di Sali di [Bis(µ3-idrosso)esacis(trifenilfosfinaoro)]. Bachelor’s Thesis.

[13] Mingos D.M. (2015). Structural and bonding patterns in gold clusters. Dalton Trans..

[14] Naldini L., Panzanelli A., Rassu G., Cariati F., Demartin F., Manassero M., Masciocchi N. (1984). Reactions of tetrahydrogenoboratebis(triphenylphosphine)copper(I) complex with nitrophenols. Inorg. Chim. Acta.

[15] Demartin F., Manassero M., Naldini L., Panzanelli A., Zoroddu M.A. (1990). Metal complexes of 2,4-diamino-5-(3′,4′,5′-trimethoxybenzyl)pyrimidine (trimethoprim) Part IV. Synthesis and X-ray structure of [CuCl(μ-OCH_3_)(trimethoprim)]_2_. Inorg. Chim. Acta.

[16] Zoroddu M.A., Naldini L., Demartin F., Masciocchi N. (1987). Metal complexes of 2,4-diamino-5-(3′,4′,5′-trimethoxybenzyl)pyrimidine (trimethoprim) and 2,4-diamino-5-(p-chlorophenyl)-6-ethylpyrimidine (pyrimethamine). Part III. Syntheses and x-ray structures of [Rh_2_(O_2_CCH_3_)_4_(trimethoprim)_2_]·2C_6_H_6_·CH_3_OH and [Rh_2_(O_2_CCH_3_)_4_(pyrimethamine)_2_]. Inorg. Chim. Acta.

[17] Demartin F., Manassero M., Naldini L., Zoroddu M.A. (1983). Metal complexes of 2,4-Diamino-5-3(3′,4′,5′-trimethoxybenzyl)pyrimidine, (trimethoprim). Part I. Synthesis and crystal structure of CoCl_2_(trimethoprim)_2_. Inorg Chim Acta.

[18] Naldini L., Zoroddu M.A., Demartin F., Manassero M. (1984). Synthesis and crystal structure of bis(p-amino-benzoate)2,2′di-pyridylcopper(II)emiaquo. Inorg. Chim. Acta.

[19] Cabras M.A., Naldini L., Zoroddu M.A., Cariati F., Demartin F., Masciocchi N., Sansoni M. (1985). Bis(triphenylphosphine)copper(I)o- and p-nitrobenzoates, synthesis and x-ray structure. Inorg. Chim. Acta.

[20] Crisponi G., Cristiani F., Nurchi V.M., Silvagni R., Ganadu M.L., Lubinu G., Naldini L., Panzanelli A. (1995). A potentiometric, spectrophotometric and 1H NMR study on the interaction of cimetidine, famotidine and ranitidine with platinum(II) and palladium(II) metal ions. Polyhedron.

[21] Bianucci A.M., Demartin F., Manassero M., Masciocchi N., Ganadu M.L., Naldini L., Panzanelli A. (1991). Metal complexes of cimetidine. Synthesis, X-ray structure determination and semiempirical calculations on the [cimetidinatecopper(II)]^+^ cation. Inorg. Chim. Acta.

[22] Naldini L., Demartin F., Manassero M., Sansoni M., Rassu G., Zoroddu M.A. (1985). Synthesis and X-ray structure of [CuPPh_3_C≡CPh]_4_, an electron deficient molecule with μ-bridging phenylacetylide ligands. J. Organomet. Chem..

[23] Malatesta L., Naldini L., Simonetta G., Cariati F. (1965). Triphenylphosphine–gold(0)/gold(I) compounds. Chem. Commun..

[24] Malatesta L., Naldini L., Simonetta G., Cariati F. (1966). Triphenylphosphine-gold(0)/gold(I) compounds. Coord. Chem. Rev..

[25] McPartlin M., Mason R., Malatesta L. (1969). Novel cluster complexes of gold(0)–gold(I). J. Chem. Soc. D.

[26] Cariati F., Naldini L., Simonetta G., Malatesta L. (1967). Clusters of gold compounds with 1,2Bis(diphenylphosphino)ethane. Inorg. Chim. Acta.

[27] Bellon P.L., Cariati F., Manassero M., Naldini L., Sansoni M. (1971). Novel gold clusters. Preparation, properties, and X-ray structure determination of salts of octakis(triarylphosphine)enneagold, [Au_9_L_8_]X_3_. J. Chem. Soc. D.

[28] Cariati F., Naldini L. (1972). Preparation and properties of gold atom cluster compounds: Octakis-(triarylphosphine)enneagold trianion. J. Chem. Soc. Dalton Trans..

[29] Adnan R.H., Andersson G.G., Polson M.I.J., Metha G.F., Golovko V.B. (2015). Factors influencing the catalytic oxidation of benzyl alcohol using supported phosphine-capped gold nanoparticles. Catal. Sci. Technol..

[30] Manassero M., Naldini L., Sansoni M. (1979). A new class of gold cluster compounds. Synthesis and X-ray structure of the octakis(triphenylphosphinegold) dializarinsulphonate, [Au_8_(PPh_3_)_8_](aliz)_2_. J. Chem. Soc. Chem. Commun..

[31] Wen F., Englert U., Gutrath B., Simon U. (2008). Crystal structure, electrochemical and optical properties of [Au_9_(PPh_3_)_8_](NO_3_)_3_. Eur. J. Inorg. Chem..

[32] Demartin F., Manassero M., Naldini L., Ruggeri R., Sansoni M. (1981). Synthesis and X-ray characterization of an iodine-bridged tetranuclear gold cluster, di-µ-iodo-tetrakis(triphenylphosphine)-tetrahedro-tetragold. J. Chem. Soc. Chem. Commun..

[33] Bruce M.I., Duffy D.N. (1986). Chemistry of the group-1B metals. XVIII. Crystal and molecular structures of nitratotris(triphenylphosphine)silver(I), Ag(O_2_NO)(PPh_3_)_3_. Aust. J. Chem..

[34] Ganadu M.L., Naldini L., Crisponi G., Nurchi V. (1991). 1H and 13C NMR studies of (phenylethynyl) (triphenylphosphine) gold(I). Spectrochim. Acta A Mol. Biomol. Spectrosc..

[35] Demartin F., Ganadu M.L., Lubinu G., Naldini L., Panzanelli A. (1995). Synthesis, NMR study, and X-ray structure of 2-(N-methyl-N′-cyano-N″-ethylguanidino) thiotriophenylphosphinegold(I). J. Inorg. Biochem..

[36] Yang Y., Ramamoorthy V., Sharp P.R. (1993). Late transition metal oxo and imido complexes. 11. Gold(I) oxo complexes. Inorg. Chem..

[37] Briant C.E., Hall K.P., Mingos D.M.P. (1983). Synthesis and structural characterisation of [Au_6_(PPh_3_)_6_]- (NO_3_)_2_·3CH_2_Cl_2_; an edge-shared bitetrahedral gold cluster. J. Organomet. Chem..

[38] Ramamoorthy V., Wu Z., Yi Y., Sharp P.R. (1992). Preparation and decomposition of gold(I) hydrazido complexes: Gold cluster formation. J. Am. Chem. Soc..

[39] Van der Velden J.W.A., Bour J.J., Steggerda J.J., Beurskens P.T., Roseboom M., Noordik J.H. (1982). Gold clusters. Tetrakis[1,3-bis(diphenylphosphino)propane]hexagold dinitrate: Preparation, x-ray analysis, and gold-197 Moessbauer and phosphorus-31{proton} NMR spectra. Inorg. Chem..

[40] Angermaier K., Schmidbaur H. (1994). A new structural motif of gold clustering at oxide centers in the dication [Au_6_O_2_(PMe_3_)_6_]^2+^. Inorg. Chem..

[41] Hong X., Cheung K.-K., Guo C.-X., Che C.-M. (1994). Luminescent organometallic gold(I) complexes. Structure and photophysical properties of alkyl-, aryl- and µ-ethynylene gold(I) complexes. J. Chem. Soc. Dalton Trans..

[42] Battistoni C., Mattogno G., Zanoni R., Naldini L. (1982). Characterisation of some gold clusters by X-ray photoelectron spectroscopy. J. Electron. Spectros. Relat. Phenom..

[43] Nagashima E., Yoshida T., Matsunaga S., Nomiya K. (2016). The effect of counteranions on the molecular structures of phosphanegold(I) cluster cations formed by polyoxometalate (POM)-mediated clusterization. Dalton Trans..

[44] Qu X., Li Y., Li L., Wang Y., Liang J., Liang J. (2015). Fluorescent gold nanoclusters: Synthesis and recent biological application. J. Nanomat..

[45] Kang X., Zhu M. (2019). Tailoring the photoluminescence of atomically precise nanoclusters. Chem. Soc. Rev..

[46] Nonappa N. (2020). Luminescent gold nanoclusters for bioimaging applications. Beilstein J. Nanotechnol..

[47] Liu M., Tang F., Yang Z., Xu J., Yang X. (2019). Recent progress on gold-nanocluster-based fluorescent probe for environmental analysis and biological sensing. J. Anal. Methods Chem..

[48] Zeng C., Jin R. (2017). Chiral gold nanoclusters: Atomic level origins of chirality. Chem. Asian J..

[49] Agrachev M., Antonello S., Dainese T., Ruzzi M., Zoleo A., Apra E., Govind N., Fortunelli A., Sementa L., Maran F. (2017). Magnetic ordering in gold nanoclusters. ACS Omega.

[50] Häkkinen H. (2008). Atomic and electronic structure of gold clusters: Understanding flakes, cages and superatoms from simple concepts. Chem. Soc. Rev..

[51] Raubenheimer H.G., Schmidbaur H. (2012). Gold chemistry guided by the isolobality concept†. Organometallics.

[52] Tian Z., Liu W., Cheng L. (2015). Progress of the experimental and theoretical studies on Au_m_(SR)_n_ clusters. Prog. Chem..

[53] Du Y., Sheng H., Astruc D., Zhu M. (2020). Atomically precise noble metal nanoclusters as efficient catalysts: A bridge between structure and properties. Chem. Rev..

[54] Zhang B., Chen J., Cao Y., Chai O.J.H., Xie J. (2021). Ligand design in ligand-protected gold nanoclusters. Small.

[55] Sousa L.M., Vilarinho L.M., Ribeiro G.H., Bogado A.L., Dinelli L.R. (2017). An electronic device based on gold nanoparticles and tetraruthenated porphyrin as an electrochemical sensor for catechol. R. Soc. Open Sci..

[56] Diao J.J., Cao Q. (2011). Gold nanoparticle wire and integrated wire array for electronic detection of chemical and biological molecules. AIP Adv..

[57] Hutchings G.J., Edwards J.K., Johnston R.L., Wilcoxon J.P. (2012). Chapter 6—Application of gold nanoparticles in catalysis. Frontiers of Nanoscience.

[58] Astruc D. (2020). Introduction: Nanoparticles in catalysis. Chem. Rev..

[59] Thompson D.T. (2007). Using gold nanoparticles for catalysis. Nano Today.

[60] Chhour P., Naha P.C., Cheheltani R., Benardo B., Mian S., Cormode D.P., Lu Z.-R., Sakuma S. (2016). Gold nanoparticles for biomedical applications: Synthesis and in vitro evaluation. Nanomaterials in Pharmacology.

[61] Cabuzu D., Cirja A., Puiu R., Grumezescu M.A. (2015). Biomedical applications of gold nanoparticles. Curr. Top. Med. Chem..

[62] Elahi N., Kamali M., Baghersad M.H. (2018). Recent biomedical applications of gold nanoparticles: A review. Talanta.

[63] Dykman L., Khlebtsov N. (2017). Gold Nanoparticles in Biomedical Applications.

[64] Medici S., Peana M., Coradduzza D., Zoroddu M.A. (2021). Gold nanoparticles and cancer: Detection, diagnosis and therapy. Semin. Cancer Biol..

[65] Wu Z., Yao Q., Chai O.J.H., Ding N., Xu W., Zang S., Xie J. (2020). Unraveling the impact of gold(I)–thiolate motifs on the aggregation-induced emission of gold nanoclusters. Angew Chem. Int. Ed. Engl..

[66] Wang E., Xu W.W., Zhu B., Gao Y. (2021). Understanding the chemical insights of staple motifs of thiolate-protected gold nanoclusters. Small.

[67] Walter M., Akola J., Lopez-Acevedo O., Jadzinsky P.D., Calero G., Ackerson C.J., Whetten R.L., Grönbeck H., Häkkinen H. (2008). A unified view of ligand-protected gold clusters as superatom complexes. Proc. Natl. Acad. Sci. USA.

[68] Pyykkö P. (2006). Understanding the eighteen-electron rule. J. Organomet. Chem..

[69] Hirai H., Takano S., Nakamura T., Tsukuda T. (2020). Understanding doping effects on electronic structures of gold superatoms: A case study of diphosphine-protected M@Au_12_ (M = Au, Pt, Ir). Inorg. Chem..

[70] De Heer W.A. (1993). The physics of simple metal clusters: Experimental aspects and simple models. Rev. Mod. Phys..

[71] Narouz M.R., Takano S., Lummis P.A., Levchenko T.I., Nazemi A., Kaappa S., Malola S., Yousefalizadeh G., Calhoun L.A., Stamplecoskie K.G. (2019). Robust, highly luminescent Au_13_ superatoms protected by N-heterocyclic carbenes. J. Am. Chem. Soc..

[72] Takano S., Hirai H., Muramatsu S., Tsukuda T. (2018). Hydride-doped gold superatom (Au_9_H)^(2+)^: Synthesis, structure, and transformation. J. Am. Chem. Soc..

[73] Maman M.P., Nair A.S., Cheraparambil H., Pathak B., Mandal S. (2020). Size Evolution dynamics of gold nanoclusters at an atom-precision level: Ligand exchange, growth mechanism, electrochemical, and photophysical properties. J. Phys. Chem. Lett..

[74] Liao L., Zhuang S., Yao C., Yan N., Chen J., Wang C., Xia N., Liu X., Li M.B., Li L. (2016). Structure of chiral Au_44_(2,4-DMBT)_26_ nanocluster with an 18-electron shell closure. J. Am. Chem. Soc..

[75] Chakraborty I., Pradeep T. (2017). Atomically precise clusters of noble metals: Emerging link between atoms and nanoparticles. Chem. Rev..

[76] Knoppe S., Bürgi T. (2014). Chirality in thiolate-protected gold clusters. Acc. Chem. Res..

[77] Pelayo J.J., Whetten R.L., Garzón I.L. (2015). Geometric quantification of chirality in ligand-protected metal clusters. J. Phys. Chem. C.

[78] Wan X.K., Yuan S.F., Lin Z.W., Wang Q.M. (2014). A chiral gold nanocluster Au_20_ protected by tetradentate phosphine ligands. Angew Chem. Int. Ed. Engl..

[79] Sugiuchi M., Shichibu Y., Konishi K. (2018). An inherently chiral Au_24_ framework with double-helical hexagold strands. Angew Chem. Int. Ed. Engl..

[80] Wang J.Q., Guan Z.J., Liu W.D., Yang Y., Wang Q.M. (2019). Chiroptical activity enhancement via structural control: The chiral synthesis and reversible interconversion of two intrinsically chiral gold nanoclusters. J. Am. Chem. Soc..

[81] Dau T.M., Chen Y.A., Karttunen A.J., Grachova E.V., Tunik S.P., Lin K.T., Hung W.Y., Chou P.T., Pakkanen T.A., Koshevoy I.O. (2014). Tetragold(I) complexes: Solution isomerization and tunable solid-state luminescence. Inorg. Chem..

[82] Hau F.K., Lee T.K., Cheng E.C., Au V.K., Yam V.W. (2014). Luminescence color switching of supramolecular assemblies of discrete molecular decanuclear gold(I) sulfido complexes. Proc. Natl. Acad. Sci. USA.

[83] Ge S., Zhao J., Ma G. (2019). Thiol stabilized extremely small gold cluster complexes with high photoluminescence. Inorg. Chem. Comm..

[84] Liu C.Y., Wei X.R., Chen Y., Wang H.F., Ge J.F., Xu Y.J., Ren Z.G., Braunstein P., Lang J.P. (2019). Tetradecanuclear and octadecanuclear Gold(I) sulfido clusters: Synthesis, structures, and luminescent selective tracking of lysosomes in living cells. Inorg. Chem..

[85] Xu W.W., Zhu B., Zeng X.C., Gao Y. (2016). A grand unified model for liganded gold clusters. Nat. Commun..

[86] Karimova N.V., Aikens C.M. (2017). Optical properties of small gold clusters Au_8_L_8_^2+^ (L = PH_3_, PPh_3_): Magnetic circular dichroism spectra. J. Phys. Chem. C.

[87] McKenzie L.C., Zaikova T.O., Hutchison J.E. (2014). Structurally similar triphenylphosphine-stabilized undecagolds, Au_11_(PPh_3_)_7_Cl_3_ and [Au_11_(PPh_3_)_8_Cl_2_]Cl, exhibit distinct ligand exchange pathways with glutathione. J. Am. Chem. Soc..

[88] Konishi K., Mingos D.M.P. (2014). Phosphine-coordinated pure-gold clusters: Diverse geometrical structures and unique optical properties/responses. Gold Clusters, Colloids and Nanoparticles I.

[89] Reddy S.T., Priver S.H., Rao V.V., Mirzadeh N., Bhargava S.K. (2018). Gold(I) and gold(III) phosphine complexes: Synthesis, anticancer activities towards 2D and 3D cancer models, and apoptosis inducing properties. Dalton Trans..

